# One-year recovery from breast cancer: Importance of tumor and treatment-related factors, resilience, and sociodemographic factors for health-related quality of life

**DOI:** 10.3389/fonc.2022.891850

**Published:** 2022-08-16

**Authors:** Katarina Veličković, Carl A. K. Borrebaeck, Pär-Ola Bendahl, Cecilia Hegardt, Per Johnsson, Corinna Richter, Lisa Rydén, Ingalill Rahm Hallberg

**Affiliations:** ^1^ Department of Psychology, Lund University, Lund, Sweden; ^2^ Department of Immunotechnology and CREATE Health Translational Cancer Center, Lund University, Lund, Sweden; ^3^ Department of Clinical Sciences Lund, Division of Oncology, Lund University, Lund, Sweden; ^4^ Department of Clinical Sciences Lund, Division of Surgery, Lund University, Lund, Sweden; ^5^ Department of Surgery, Skåne University Hospital, Lund, Sweden; ^6^ Department of Health Sciences, Lund University, Lund, Sweden

**Keywords:** breast cancer, psychological resilience, longitudinal study, health-related quality of life (HRQL), tumor characteristics, breast cancer treatment, biopsychosocial (BPS) model, multidisciplinary

## Abstract

**Aim:**

This study investigated the changes in health-related quality of life from diagnosis to 1 year after diagnosis in breast cancer (BC) patients and the influence of clinical, psychological, and sociodemographic variables. An additional aim was to explore the mediating and moderating effects of resilience on changes in health-related quality of life.

**Methods:**

A longitudinal population-based study was conducted in southern Sweden. Newly diagnosed BC patients filled in measures of health-related quality of life, resilience, and sociodemographic variables at diagnosis (*N* = 980) and 1 year post-diagnosis (*N* = 780). Clinical variables were extracted from the Swedish national breast cancer quality registry. Mixed-model analyses were performed.

**Results:**

Most health-related quality of life outcomes declined from diagnosis to 1 year post-diagnosis. Role limitations due to emotional problems remained the same, whereas mental health improved. Lower health-related quality of life outcomes were associated with symptomatic detection and axillary dissection. Patients with a higher TNM stage and histologic grade and estrogen receptor (ER)-negative and human epidermal growth factor 2 (HER2)-positive status, who received chemotherapy, antibody therapy, or bisphosphonate therapy, had a steeper decline in outcomes. Changes in resilience were positively associated with all outcomes but did not mediate or moderate changes in any. Resilience at baseline moderated changes in bodily pain, vitality, and mental health, with higher baseline resilience being associated with a steeper decline, possibly due to floor or ceiling effects. Patients with lower socioeconomic status, educational level, and older age had a lower health-related quality of life.

**Conclusion:**

Physical health-related quality of life among breast cancer patients declined 1 year post-diagnosis, whereas mental health-related quality of life improved. Low resilient patients may be especially vulnerable at diagnosis. Biopsychosocial assessment at diagnosis can help identify patients who may require additional support. A multidimensional treatment plan should be started early to help overcome the problems in everyday activities.

## Introduction

Like in most countries, breast cancer (BC) is the most common type of cancer among women in Sweden, with nearly 8,000 new cases yearly (1, 2). Starting with signs of a lump in the breast or screening detection, diagnosis, and treatment, it is an extremely stressful life event, and a long and often multimodal treatment follows, which can negatively affect their physical and psychological health (3). Considering that the wellbeing and quality of life (QoL) of BC patients are affected from diagnosis to treatment and beyond, cross-sectional studies fail to demonstrate how these evolve over time. To fully understand the recovery process, longitudinal studies covering clinical, psychological, and sociodemographic variables are needed (4). Unlike clinical and sociodemographic factors, psychological factors such as resilience and the interaction with other biopsychosocial factors have so far been understudied. A precise stratification of patients who have a higher risk for a worsened health-related quality of life (HRQoL) is needed in order to provide tailored rehabilitation plans (5–7). Hence, this study aims to provide a better ground for an understanding of the process of recovery from BC by exploring the changes in HRQoL from diagnosis to 1 year after diagnosis, as well as to determine the importance of biopsychosocial factors influencing these changes.

Variation in tumor characteristics and treatment intensity in BC is great, resulting in varying degrees of distress. Women with ductal carcinoma *in situ* and those with invasive BC might experience similar distress after surgery (8, 9). Other studies found that more advanced cancer at diagnosis predicted distress (10) and suicidal ideation after surgery (11). Hormone-sensitive tumors are common (12), and those with estrogen receptor (ER)-positive BC have longer disease-free periods and survival (13), which may indicate higher HRQoL. In contrast, 15%–20% of BCs have overexpression of the human epidermal growth factor 2 (HER2) receptors with a higher likelihood of recurrence and metastasis and a lower disease-free period (14–16). However, recent advances in treatment for HER2-positive BC (e.g., trastuzumab and trastuzumab emtansine) have led to increased survival (17–19). Additionally, in HRQoL research, it is difficult to separate the effect of hormonal status and other tumor characteristics from accompanying treatments.

Considering the variety of BC treatment modalities, it is important to explore the potentially varying impact they may have on recovery. Surgery is the most common treatment strategy for BC and has been associated with less body satisfaction and lower physical HRQoL (20). Full mastectomy may lead to more side effects and worse body image compared to breast-conserving surgery (21, 22). Axillary lymph node dissection may indicate a spread of cancer to the patient and is associated with arm pain, swelling, lymphedema, and numbness (23), as compared to sentinel lymph node biopsy (SLNB), which comprises fewer complications. Chemotherapy has been shown as an especially great risk factor for distress as compared to other modalities (10, 20, 24). Endocrine treatment often encompasses uncomfortable side effects such as hot flashes, loss of sexual function, and weight gain (12, 25). Antibody therapy, a treatment option for HER2-positive BC (26), can have serious but often short-term adverse effects, such as cardiotoxicity (27). A recent review of bisphosphonate drugs highlighted various side effects including bone and back pain, fatigue, and neurosensory problems (28). Thus, the recovery may differ due to treatment and its side effects.

In line with the biopsychosocial model, the influence of psychological factors such as psychological resilience should be considered when investigating BC recovery. It refers to a positive adaptation to significant adversity (29). There is a lack of agreement on whether resilience is a stable construct or if it is susceptible to change, especially following life-altering events. Some studies have uncovered biological markers of resilience (30), providing support for the stable nature of resilience. Other studies focus on social and environmental constituents of resilience, which are susceptible to change (31). Research has mainly been on treatment and survivorship phases, and resilience was often measured through its associated traits, such as optimism or hope, or as an outcome (3). For example, greater internal strength at baseline has been associated with reduced distress and an enhanced QoL at follow-up (32). Optimism at diagnosis contributed to lower distress at follow-up (33). Baseline optimism and hope have been associated with QoL during BC survivorship (34). Cross-sectional studies measuring resilience as a construct have been associated with higher QoL and lower distress among BC patients (35, 36). Recently, resilience has been associated with HRQoL 1 year after diagnosis (37). Research on resilience in social science is dominated (80%) by cross-sectional (38) designs, uncovering the need for longitudinal investigations. It is thus uncertain whether resilience changes after BC diagnosis and treatment and if this change in resilience may serve as a mechanism of recovery, mediating the process of recovery, or if it serves as a moderator of recovery, i.e., if more resilient patients have a faster recovery.

Lastly, certain sociodemographic characteristics have been found to predict distress in BC patients. Young age and low socioeconomic status have consistently been associated with worse outcomes (10). Living alone was associated with a lower QoL (39) and more depressive symptoms (40, 41) in a BC population. A negative association between social support and BC progression has been consistently found across studies (42), with living alone potentially indicating less social support. Still, it is unclear whether patients from vulnerable sociodemographic categories also experience a slower recovery process.

The main aim of the study was therefore to determine how HRQoL changes from diagnosis to 1 year after diagnosis among BC patients, while exploring the influence of clinical, psychological, and sociodemographic variables and their potential moderating effects on these changes. An additional aim of this study was to determine whether changes in resilience over time mediate or moderate the change in HRQoL in BC patients from diagnosis to 1 year post-diagnosis, as well as whether resilience at diagnosis is associated with the change in HRQoL over time.

## Materials and methods

### Context and participants

The current study is a population-based, prospective longitudinal study on newly diagnosed patients with primary BC recruited from the Helsingborg Hospital, Blekinge County Hospital, Hallands Hospital Halmstad, and Central Hospital Växjö. The sample was obtained within the larger study, SCAN-B Resilience (Clinical Trials number: NCT03430492) (43), as part of the Sweden Cancerome Analysis Network—Breast (SCAN-B; Clinical Trials number: NCT02306096) (44). Inclusion criteria for the study were 1) being newly diagnosed with primary BC, 2) oral and written agreement to participate in the study at one of the abovementioned study sites, 3) being over 18 years old, and 4) understanding the Swedish language. The main reasons given for declining to participate included shock from diagnosis, physical and mental health problems, and language issues (44).

### Procedure

Data collection occurred on the same day as being informed about the BC diagnosis and discussing the treatment plan with a physician. The inclusion of the patients normally took place 2 to 3 weeks after the diagnostic workup, screening procedures, and biopsy. Patients were informed about the study and asked to participate, after which they filled in the consent form. Participation was voluntary, responses were pseudonymized, and participants could withdraw participation at any time. Patients filled in the measures of resilience, HRQoL, and sociodemographic measures electronically or on paper. One year after diagnosis, participants who took part at baseline and were still alive were sent a letter asking to participate in the follow-up study. Follow-up data were also collected electronically or on paper, at participants’ homes, and included measures of resilience and HRQoL.

### Measures

#### Health-related quality of life

The Short Form 36 Health Survey (SF-36) (45) is a self-report measure of HRQoL. It contains 35 items, which correspond to eight domains: physical functioning, role limitations due to physical problems, role limitations due to emotional problems, bodily pain, general health, vitality, social functioning, and mental health. Two additional scores are obtained from the eight domains: physical health component score (PCS) and mental health component score (MCS). Scores are calculated using PRO CoRE software (46) and range from 0 to 100. Norm scores exist for the general Swedish population (47). Higher scores indicate better functioning. All subscores had at least acceptable internal consistency at both time points in the current study (α > 0.70). One additional item in the scale assesses perceived change in current health compared to 1 year prior.

#### Clinical variables

Clinical variables were menstrual status, mode of detection, TNM stage, ER status, HER2 status, histologic grade, primary therapy, type of surgery, time of reconstruction, axillary surgery, adjuvant chemotherapy, adjuvant endocrine therapy, adjuvant bisphosphonate therapy, adjuvant antibody therapy, and adjuvant radiotherapy. All clinical variables were extracted from the Swedish national breast cancer quality registry (NKBC) registry, the national BC registry where new cases of primary BC are registered, along with tumor and treatment characteristics (48). Tumor data concern the largest tumor found, although the number of tumors is also registered. Inclusion of data in the registry is optional, but close to 100% of all cases are registered. The registry is updated continuously. Variable categories are displayed in **Table 1**.

**Table 1:** Baseline characteristics of participants included at follow-up (respondents) and those who dropped out (*n* = 190) or died (*n* = 10) before follow-up (non-respondents).

Measures	Respondents *N* = 780^a^ (79.6%)	Non-respondents *N* = 200^a^ (20.4%)	*p*	
Gender			.21	
Female	775 (99.4%)	197 (98.5%)		
Male	5 (0.6%)	3 (1.5%)		
Age at diagnosis, mean (SD)	62.92 (11.01)	61.45 (12.69)	.13	
Education level			.51	
Primary school <9 years	90 (11.6%)	28 (14.3%)		
Primary school completed	102 (13.2%)	24 (12.2%)		
Upper secondary education	152 (19.6%)	40 (20.4%)		
Postsecondary school <2 years	87 (11.2%)	16 (8.2%)		
Postsecondary school >2 years	330 (42.6%)	87 (44.4%)		
PhD (doctoral education)	14 (1.8%)	1 (0.5%)		
Living situation			.30	
Alone	168 (21.5%)	54 (27.1%)		
With children <18	18 (2.3%)	6 (3%)		
With children <18 and other adults	91 (11.7%)	24 (12.1%)		
With other adults	503 (64.5%)	115 (57.8%)		
Socioeconomic status			.03*	
Able to pay unexpected bill	711 (91.4%)	170 (86.3%)		
Unable to pay unexpected bill	67 (8.6%)	27 (13.7%)		
Mode of detection			.38	
Symptomatic	299 (40.1%)	82 (43.6%)		
Screening	446 (59.9%)	106 (56.4)		
Menstrual status			.16	
Premenopausal	124 (18.3%)	38 (23.2%)		
Postmenopausal	553 (81.7%)	126 (76.8%)		
Type of cancer			.15	
Invasive cancer	652 (91.7%)	158 (88.3%)		
Carcinoma *in situ*	59 (8.3%)	21 (11.7%)		
TNM stage			.97	
Stage 0	37 (5%)	14 (7.6%)		
Stage I	471 (64.2%)	110 (59.5%)		
Stage II	218 (29.7%)	58 (31.4%)		
Stage III	8 (1.1%)	3 (1.6%)		
Histologic grade			.74	
Grade I	128 (18%)	38 (22.2%)		
Grade II	389 (54.8%)	76 (44.4%)		
Grade III	193 (27.2%)	57 (33.3%)		
ER status			.81	
ER negative	86 (13.2%)	20 (12.5%)		
ER positive	564 (86.8%)	140 (87.5%)		
HER2 status			.22	
HER2 negative	572 (88.3%)	143 (91.7%)		
HER2 positive	76 (11.7%)	13 (8.3%)		
Primary therapy			.06	
Systemic therapy	43 (5.5%)	18 (9.7%)	
Surgery	697 (94.2%)	168 (90.3%)	
Type of surgery			.23	
Partial mastectomy	536 (72%)	126 (67%)		
Full mastectomy	203 (27.3%)	59 (31.4%)		
Subcutaneous mastectomy	5 (0.7%)	3 (1.6%)		
Immediate reconstruction			.76	
No	372 (93.2%)	59 (92.2%)		
Yes	27 (6.8%)	5 (7.8%)		
Type of axillary surgery			.38	
Only sentinel nodes	569 (77.6%)	146 (80.7%)		
Only axillary dissection	72 (9.8%)	19 (10.5%)		
Sentinel nodes and axillary dissection	92 (12.6%)	16 (8.8%)		
Radiotherapy			.22	
No	123 (17.9%)	35 (22%)		
Yes	566 (82.1%)	124 (78%)		
Endocrine therapy			.28	
No	225 (32.7%)	59 (37.1%)		
Yes	464 (67.3%)	100 (62.9%)		
Chemotherapy			.10	
No	452 (65.6%)	115 (72.3%)		
Yes	237 (34.4%)	44 (27.7%)		
Bisphosphonate therapy			.07	
No	556 (81.8%)	138 (87.9%)		
Yes	124 (18.2%)	19 (12.1%)		
Antibody therapy			.34	
No	615 (89.3%)	146 (91.8%)		
Yes	74 (10.7%)	13 (8.2%)		
CD-RISC, mean (SD)	70.73 (12.59)	71.90 (12.45)	.24	
SF-36, mean (SD)				
Physical functioning	85.92 (18.23)	81.85 (21.36)	.01*	
Role limitations—physical	83.52 (32.99)	78.72 (35.11)	.08	
Bodily pain	82.04 (20.42)	77.70 (22.94)	.02*	
General health perceptions	71.30 (18.95)	68.76 (19.82)	.10	
Vitality	67.71 (22.53)	65.38 (21.45)	.19	
Social functioning	85.03 (21.39)	81.54 (23.34)	.06	
Role limitations—emotional	77.38 (36.44)	76.58 (36.82)	.79	
Mental health	70.57 (20.68)	69.50 (20.51)	.52	
PCS	53.90 (8.13)	51.97 (8.85)	.004**	
MCS	48.32 (11.81)	48.00 (11.61)	.74	
Perceived change in health			.41	
Much better than a year ago	50 (6%)	14 (7%)		
Somewhat better than a year ago	87 (11%)	20 (10%)		
About the same	503 (65%)	118 (60%)		
Somewhat worse than a year ago	116 (15%)	39 (20%)		
Much worse than a year ago	21 (3%)	6 (3%)		

ER, estrogen receptor; HER2, human epidermal growth factor 2; CD-RISC, Connor-Davidson Resilience Scale; SF-36, Short Form 36 Health Survey; PCS, physical health component score; MCS, mental health component score.

a In some cases, the sum is smaller than 780 for respondents and 200 for non-respondents due to missing data.

*p <.05.

**p <.01.

#### Resilience

Resilience was measured using the Swedish version of the Connor-Davidson Resilience Scale (CD-RISC) (49). The scale is the most widely used self-report scale to measure resilience and contains 25 items evaluated on a 5-point Likert scale from 0 (‘Not true at all’) to 4 (‘True nearly all the time’). A total score is calculated, ranging from 0 to 100, with higher scores indicating higher resilience. Internal consistency of the scale was high both at baseline and follow-up (α > 0.90).

#### Sociodemographic variables: study-specific questionnaire

Participants reported their age, gender, highest level of education, socioeconomic status (SES), and living arrangement at baseline in the study-specific questionnaire. Socioeconomic status was indicated by answering whether they would be able to pay an unexpected bill of SEK 11,000. Response categories are presented in **Table 1**.

### Data analyses

To address the research questions, mixed-model regression analysis was employed. Mixed models are statistical models used to analyze data on more than one level of analysis (50). In the case of our study, mixed models were applied to multiple observations nested within a person, the primary objective being to model the predictors of change over time. Thus, there were two levels of data. The first one was the within-subject factor called *time*, which referred to the estimations of 10 SF-36 subscores and CD-RISC at baseline and 1-year follow-up. The second level was between-subject factors, which included clinical and sociodemographic variables as potential predictors and moderators of the change in SF-36 subscores. A potential moderator was considered a variable that altered the relationship between time and an SF-36 subscore. A possible moderating effect was thus found if there was an interaction between *time* and a variable of interest. All factors were estimated as fixed factors.

Missing data on CD-RISC were handled by calculating the mean of the remaining items and multiplying by 25 for those missing four or fewer items. For participants with four or fewer missing items on SF-36, mean imputation was performed, as only less than 5% of subjects had missing data (51). Imputation did not result in a decrease in variability in the data. Additionally, 140 participants had missing values for at least one clinical variable. There were no differences between participants with one or more missing values for the clinical variables and those with complete clinical variables on any other study variables (*p*’s >.05), indicating the data were missing completely at random.

Characteristics of participants who responded to the follow-up study and those who only took part at baseline were compared using t-tests for independent samples, χ^2^ tests of independence, and the Mantel–Haenszel test of the trend for ordinal variables. Due to the very small number of participants who died prior to follow-up (*n* = 10), they were analyzed together with the subgroup of patients who decided to drop out of the study. The change over time in the perceived change in current health compared to 1 year prior was assessed using the Wilcoxon signed-rank test due to the ordinal nature of the item.

Mixed-model analyses were performed to explore the changes in HRQoL from baseline to follow-up. First, only time and random effects of intercepts (i.e., variability in intercepts of individual units) were included in the model. After that, the potential main and moderating effects of biopsychosocial variables were investigated. All these variables were investigated in separate models, which included the main effect of time, the main effect of the biopsychosocial variable, the interaction between time and the biopsychosocial variable, potential covariates, and random intercepts. It was found that in all models, a large amount of variance could be explained by between-patient variability, i.e., the difference in intercepts of individual units.

The role of menstrual status and the influence of mode of detection and *tumor characteristics* on changes in HRQoL was explored while adjusting for age for the latter two. Tumor characteristics included TNM stage, histologic grade, ER status, and HER2 status. To facilitate interpretation of whether tumor characteristics play a role above strenuous treatment, they were adjusted for radiotherapy, chemotherapy, bisphosphonate, endocrine, and antibody therapy. As it is very difficult to separate ER status from endocrine therapy, as well as HER2 status from antibody therapy, ER status was not adjusted for endocrine therapy, and HER2 status was not adjusted for antibody therapy. The influence of *treatment modalities*, including the type of primary therapy, type of surgery, axillary dissection, radiotherapy, chemotherapy, endocrine, antibody, and bisphosphonate therapy, was then tested while adjusting for age. For the type of surgery, the category of subcutaneous mastectomy was excluded from the analysis due to too few respondents (*n* = 5). The time of breast reconstruction was not analyzed due to a small subsample with immediate reconstruction (*n* = 27).

Resilience was then investigated as a covariate. The possible mediation was observed if the change in resilience explained the change in HRQoL subscores over time, i.e., it was established when the effect of time adjusted for resilience was weaker than the unadjusted effect of time. To test whether resilience at baseline influenced changes in HRQoL, resilience at baseline was included as a covariate. Finally, the main and moderating effects of SES, living situation, and education level were investigated. All were adjusted for age and living situation, and education level was adjusted for SES.

Differences in categories were further investigated using *post-hoc* tests utilizing the Bonferroni correction for multiple comparisons (52). Significant interactions were explored using simple slopes, with −1SD, mean, and +1SD levels of the continuous moderators, with slopes being adjusted for covariates. All covariates were mean-centered to enhance the interpretability of findings. Analyses were performed using RStudio Software, version 1.1.456 (53), and jamovi Software, version 1.6 (54).

## Results

**Figure 1:**
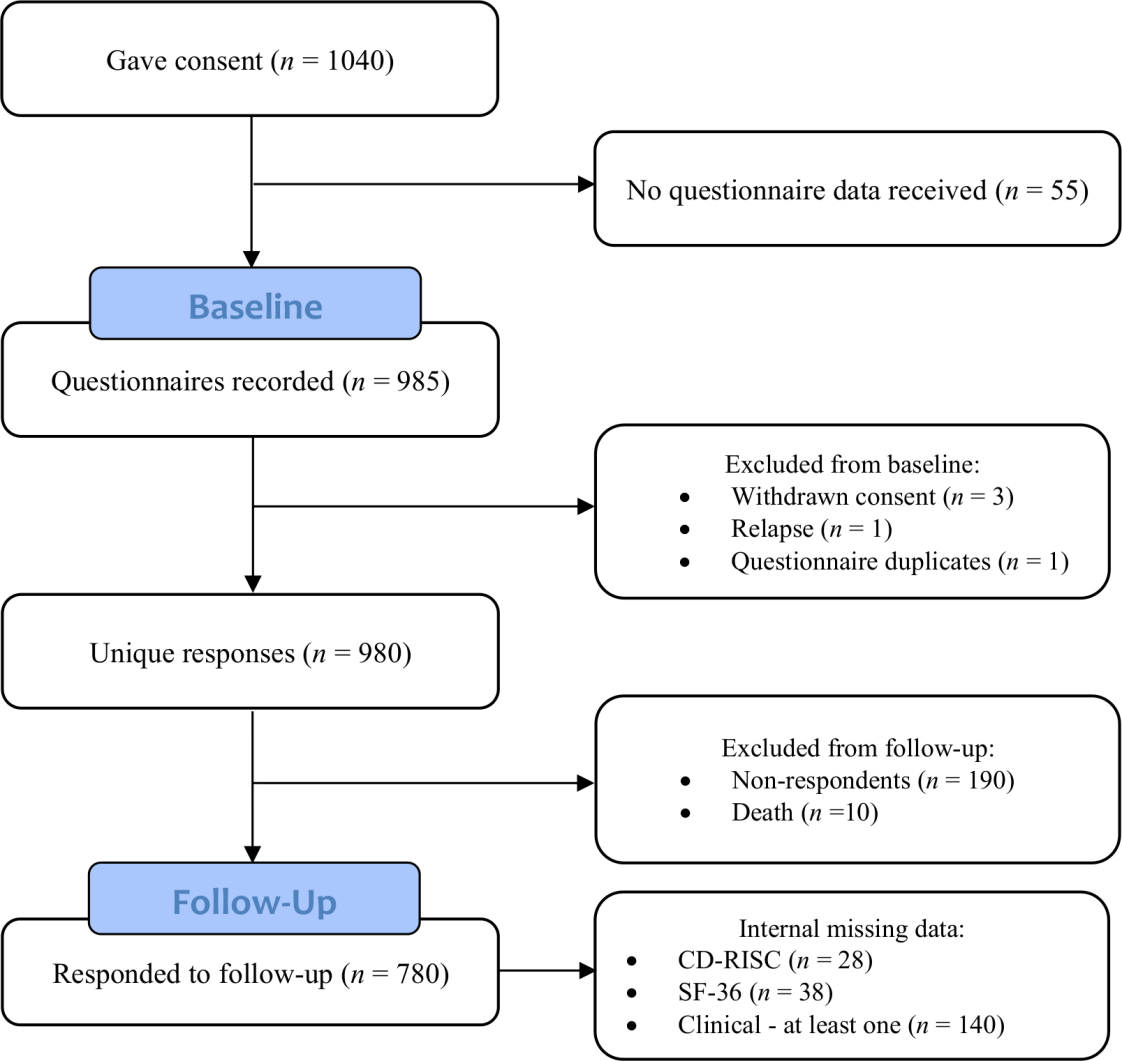
Flow diagram of the study cohort.

In total, 980 participants took part in the study at baseline (**Figure 1**). Out of the total sample, eight participants (0.8%) were male, whereas the rest were female. Participants were aged between 31 and 89 years (*M* = 62.6, *SD* = 11.4) at the time of diagnosis. At follow-up, the sample included 780 participants. Non-respondents to follow-up (i.e., those who dropped out and patients who died; **Figure 1**) were more likely to be in the lower socioeconomic group, had worse physical functioning, had more bodily pain, and had a lower physical health summary score at baseline as compared to respondents (**Table 1**). Respondents and non-respondents did not differ on any other variables included in the study at baseline. Correlations, means, and standard deviations of resilience and HRQoL subscores at baseline and follow-up are presented in the **Supplementary Material**.

### Changes in health-related quality of life over time

All but three HRQoL subscales declined over time (**Table 2**), indicating a functional decline. Role limitations remained the same, while mental health and MCS improved. The biggest decline was found for PCS, followed by bodily pain and physical functioning. Additionally, participants’ perceptions of their current health as compared to 1 year earlier changed from baseline to follow-up (Z = 5.1, *p* <.001). Participants estimated to a greater extent that their health at baseline was worse than at 1 year prior than at follow-up.

**Table 2:** HRQoL scores at baseline and 1-year follow-up (*N =* 760).

	Time F	df1^a^	df2^a^	Time *p*	M1^b^	M2^b^
Physical functioning	137.77	1	759	<.001	86.35	80.95
Role limitations—physical	98.45	1	759	<.001	84.24	69.05
Bodily pain	213.87	1	759	<.001	82.09	70.31
General health	22.80	1	759	<.001	71.51	68.46
Vitality	61.36	1	759	<.001	67.97	61.93
Social functioning	9.15	1	759	.003	85.33	82.66
Role limitations—emotional	0.00	1	759	.950	77.94	77.85
Mental health	76.35	1	759	<.001	70.74	77.07
Physical health component	356.73	1	759	<.001	54.02	48.73
Mental health component	30.11	1	759	<.001	48.43	50.75

HRQoL, health-related quality of life.

a Degrees of freedom.

b Estimated marginal means at baseline (M1) and follow-up (M2).

### Clinical variables and health-related quality of life

**Figure 2:**
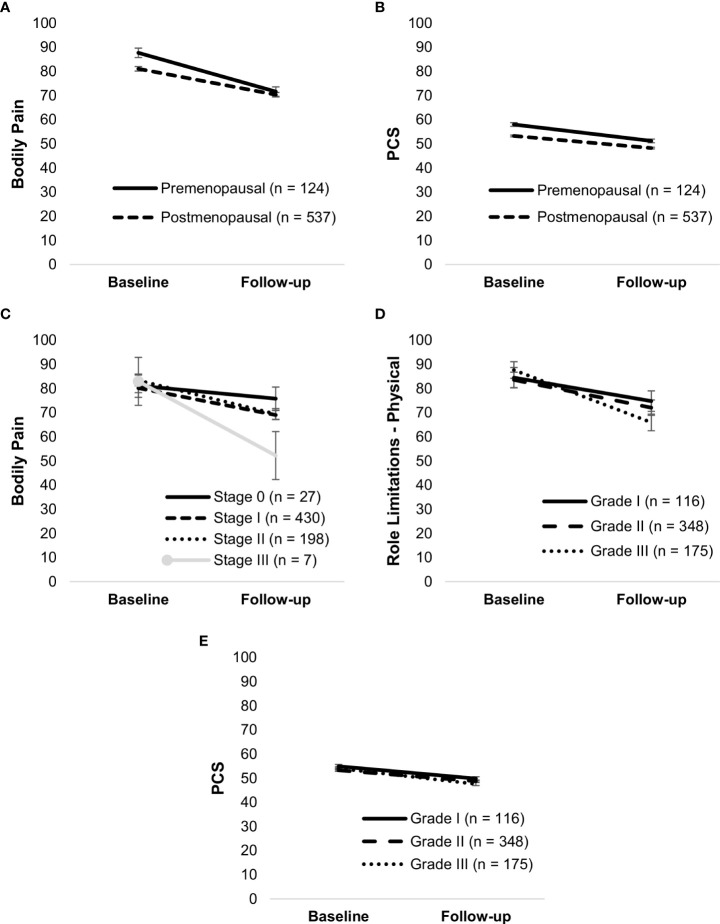
Simple slopes of the moderating effect of menstrual status on changes in bodily pain **(A)** and PCS **(B)**; TNM stage on changes in bodily pain **(C)**; histologic grade on changes in physical role limitations **(D)** and PCS **(E)**. Higher scores on outcome variables indicate better functioning. Error bars show standard errors. Simple slopes for TNM stage and histologic grade are adjusted for age, radiotherapy, and systemic therapy. PCS, physical health component score.

Symptomatic mode of detection and postmenopausal status were associated with worse HRQoL. Patients who were symptomatic had lower scores on all HRQoL outcomes than those diagnosed through screening (*p*’s <.05), except for physical functioning (*p* = .05). Mode of detection did not moderate any changes in HRQoL. Postmenopausal women had worse physical functioning, PCS, and bodily pain, and better MCS (all *p*’s <.05) as compared to premenopausal women. Menstrual status moderated the changes in bodily pain, F(1,659) = 5.90, *p* = .01, and PCS, F(1,659) = 5.17, *p* = .02. Although both premenopausal and postmenopausal women had a decline in bodily pain and PCS (*p*’s <.001), the decline was steeper in premenopausal women (**Figures 2A, B**). It is important to note that significant *p*-values may reflect a large sample size.

#### Tumor characteristics

Tumor characteristics such as ER-negative and HER2-positive tumors, higher TNM stage, and histologic grade were associated with a steeper decline in certain HRQoL outcomes. TNM stage moderated bodily pain, F(3,658) = 3.13, *p* = .02. Those with stage I, *t*(658) = 10.40, *p* <.001, stage II, *t*(658) = 8.78, *p* <.001, and stage III, *t*(658) = 3.65, *p* <.001, had a decline in bodily pain, whereas those with stage 0 BC (ductal carcinoma *in situ*) maintained stable levels, *t*(658) = 1.25, *p* = .21 (**Figure 2C**). Histologic grade moderated physical role limitations, F(2,636) = 4.21, *p* = .01, and PCS, F(2,636) = 3.80, *p* = .02. Participants with grades I, II, or III showed a decline in role limitations due to physical problems and PCS over time, but it was steeper in those with grade III (*p*’s <.05) (**Figures 2D, E**).

**Figure 3:**
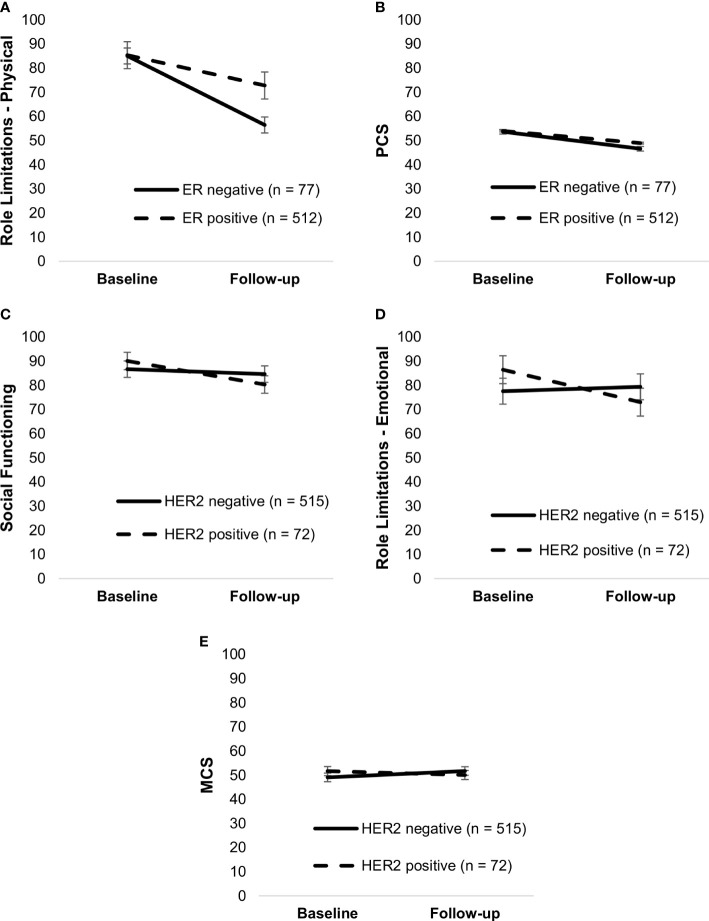
Simple slopes of the moderating effect of ER status on changes in role limitations—physical **(A)** and PCS **(B)**; HER2 status on changes in social functioning **(C)**, role limitations—emotional **(D)**, and MCS **(E)**. Higher scores on outcome variables indicate better functioning. Error bars show standard errors. Simple slopes are adjusted for age, radiotherapy, and systemic therapy. PCS, physical health component score; MCS, mental health component score; HER2, human epidermal growth factor 2.

ER-negative patients had worse limitations due to physical problems, F(1,582) = 4.72, *p* = .03, and emotional problems, F(1,582) = 6.57, *p* = .01, as compared to ER-positive patients, after adjusting for radiotherapy, chemotherapy, bisphosphonate therapy, and antibody therapy. Additionally, ER status moderated role limitations due to physical problems, F(1,587) = 9.90, *p* = .002, and PCS, F(1,587) = 4.54, *p* = .03, with ER-positive patients having a steeper decline in role limitations and PCS (*p*’s <.001) (**Figures 3A, B**). HER2 status moderated social functioning, F(1,585) = 6.65, *p* = .01, emotional role limitations, F(1,585) = 8.70, *p* = .003, and MCS, F(1, 585) = 8.78, *p* = .003, after adjusting for radiotherapy, chemotherapy, bisphosphonate therapy, and endocrine therapy (**Figures 3C–E**). HER2-positive participants experienced a decline in social functioning, *t*(585) = 3.48, *p* <.001, limitations due to emotional problems, *t*(585) = 2.77, *p* = .006, and no change in MCS, *t*(585) = 1.21, *p* = .23. HER2-negative participants experienced no change in social functioning, *t*(585) = 1.95, *p* = .051, and in role limitations due to emotional problems, *t*(585) = 1.00, *p* = .32, but an increase in MCS, *t*(585) = 5.23, *p* <.001.

#### Treatment characteristics

**Figure 4:**
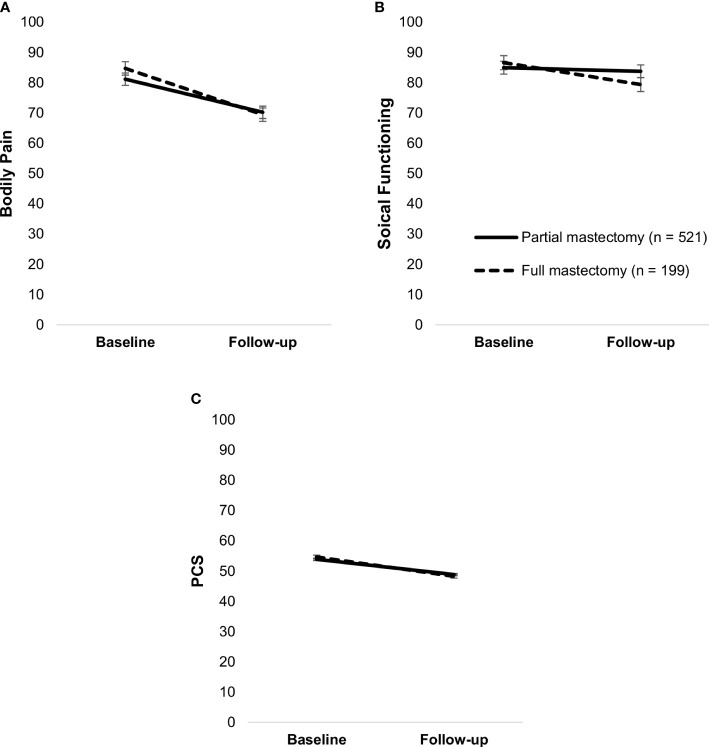
Simple slopes of the moderating effect of type of surgery on changes in bodily pain **(A)**, social functioning **(B)**, and PCS **(C)**. Higher scores on outcome variables indicate better functioning. Error bars show standard errors. Simple slopes are adjusted for age. PCS, physical health component score.

Certain treatment modalities, including axillary dissection, full mastectomy, chemotherapy, antibody therapy, and bisphosphonate therapy, were associated with worse HRQoL or a steeper decline in HRQoL over time. Whether patients received surgery or systemic therapy as primary therapy was not associated with any HRQoL measures (all *p*’s >.05). Type of surgery moderated bodily pain, F(1,718) = 5.48, *p* = .02, and PCS, F(1,718) = 4.24, *p* = .04; those who had a full mastectomy had a steeper decline than those with a partial mastectomy (*p*’s <.001). It also moderated social functioning, F(1,718) = 8.73, *p* = .003, with patients who had a full mastectomy having declined in social functioning, *t*(718) = 3.82, *p* <.001, and those with a partial mastectomy remaining stable over time, *t*(718) = 1.12, *p* = .26 (**Figure 4**).

Patients who received only SLNB had better role limitations due to physical problems, F(2,711) = 10.06, *p* <.001, physical functioning, F(2,711) = 3.17, *p* = .04, and PCS, F(2,617) = 4.29, *p* = .01, than those who received axillary dissection. Patients who received radiotherapy had worse general health than those who did not, F(1,675) = 4.32, *p* = .04. Radiotherapy was not associated with any other HRQoL outcomes (*p*’s >.05).

##### Systemic therapy

**Figure 5:**
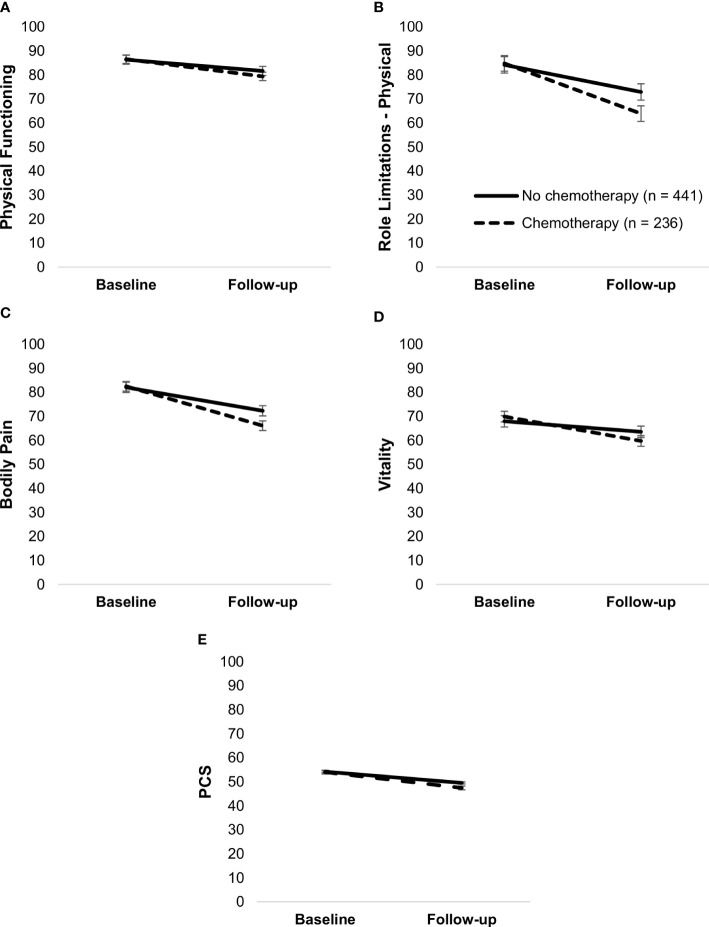
Simple slopes of the moderating effect of chemotherapy on changes in physical functioning **(A)**, Role limitations due to physical problems **(B)**, bodily pain **(C)**, vitality **(D)**, and PCS **(E)**. Higher scores on outcome variables indicate better functioning. Error bars show standard errors. Simple slopes are adjusted for age. PCS, physical health component score.

Endocrine therapy was not associated with any HRQoL measures (all *p*’s >.05). Chemotherapy, however, moderated changes in physical functioning, F(1,675) = 5.94, *p* = .01, role limitations due to physical problems, F(1,675) = 8.99, *p* = .003, bodily pain, F(1,675) = 13.70, *p* <.001, vitality, F(1,675) = 11.71, *p* <.001, and PCS, F(1,675) = 8.96, *p* = .003 (**Figure 5**). Although there was a decline in both patients who received chemotherapy and those who did not (*p*’s <.001), the decline was steeper in the former group.

**Figure 6:**
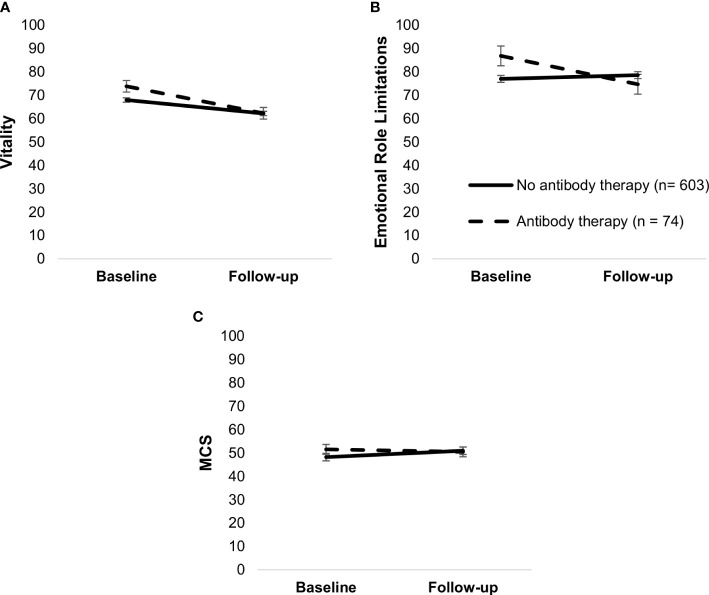
Simple slopes of the moderating effect of antibody therapy on changes in vitality **(A)**, role limitations—emotional **(B)**, and MCS **(C)**. Higher scores on outcome variables indicate better functioning. Error bars show standard errors. Simple slopes are adjusted for age. MCS, mental health component score.

Antibody therapy moderated changes in vitality, F(1,675) = 5.03, *p* = .02, role limitations due to emotional problems, F(1,675) = 7.35, *p* = .01, and MCS, F(1,675) = 7.14, *p* = .01. Patients who received antibody therapy had a steeper decline in vitality (both *p*’s <.001) and role limitations due to emotional problems, *t*(675) = 2.54, *p* = .01, and unchanged MCS, *t*(675) = 0.80, *p* = .42, whereas those who did not receive it had unchanged role limitations, *t*(675) = 0.95, *p* = .34, and increased MCS, *t*(675) = 5.80, *p* <.001 (**Figure 6**).

**Figure 7:**
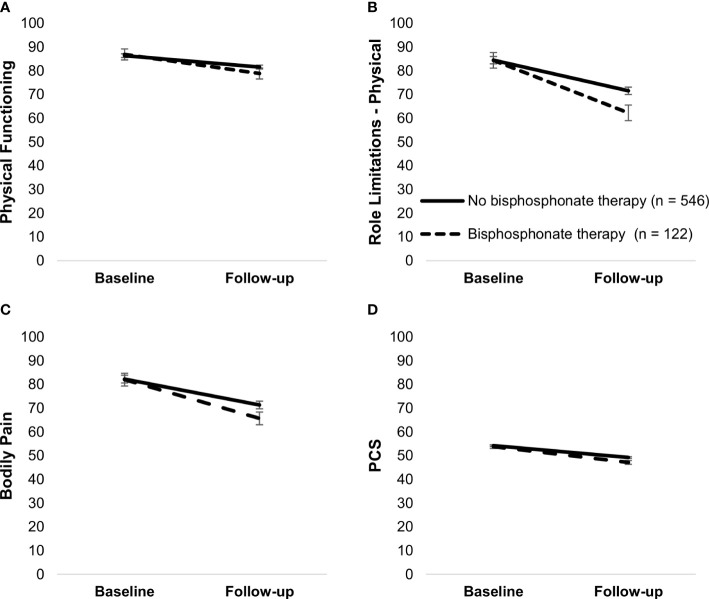
Simple slopes of the moderating effect of bisphosphonate therapy on changes in physical functioning **(A)**, role limitations—physical **(B)**, bodily pain **(C)**, and PCS **(D)**. Higher scores on outcome variables indicate better functioning. Error bars show standard errors. Simple slopes are adjusted for age. PCS, physical health component score.

Bisphosphonate therapy was associated with more bodily pain, F(1,665) = 4.45, *p* = .03, as well as moderated physical functioning, F(1,666) = 6.64, *p* = .01, role limitations due to physical problems, F(1,666) = 4.64, *p* = .03, bodily pain, F(1,666) = 6.03, *p* = .01, and PCS, F(1,666) = 4.43, *p* = .04. Although patients who received bisphosphonate therapy and those who did not had a decline in these outcomes (*p*’s <.001), those who received bisphosphonate therapy had a steeper decline (**Figure 7**).

### Resilience and health-related quality of life

Changes in resilience were positively associated with changes in all HRQoL outcomes over time (**Table 3**). Resilience had the strongest association with MCS and general health. The associations were the weakest for PCS and bodily pain. Resilience did not moderate changes in any HRQoL measures, indicating that HRQoL did not improve or deteriorate more strongly over time for those with higher or lower resilience. The effect of time adjusted for resilience (**Table 3**) was not weaker than the unadjusted effect of time (**Table 2**), indicating that changes in resilience did not mediate the changes in HRQoL.

**Table 3:** Resilience as a within-person covariate to HRQoL (*N =* 760).

	Time			Resilience			Time * Resilience						
	F^a^	*p*		F^b^	*p*		F^c^	*p*		M1^d^	M2^d^	
Physical functioning	121.24	<.001		55.78	<.001		0.29	.59		86.18	81.13	
Role—physical	88.17	<.001		55.41	<.001		0.09	.76		83.85	69.47	
Bodily pain	201.13	<.001		42.74	<.001		1.07	.30		81.85	70.50	
General health	13.23	<.001		216.45	<.001		1.08	.29		71.13	68.88	
Vitality	47.58	<.001		182.57	<.001		0.96	.33		67.51	62.35	
Social functioning	5.13	.02		113.87	<.001		0.08	.78		84.98	83.02	
Role—emotional	0.51	.47		119.91	<.001		0.07	.79		77.37	78.44	
Mental health	107.86	<.001		266.91	<.001		0.00	.98		70.30	77.51	
PCS^e^	337.66	<.001		27.18	<.001		0.00	.99		53.96	48.80	
MCS^f^	47.52	<.001		229.66	<.001		0.01	.92		48.19	50.99	

HRQoL, health-related quality of life.

a df = 1, 761.

b df = 1, 1445.

c df = 1, 797.

d Estimated marginal means at baseline (M1) and follow-up (M2).

e Physical health component score.

f Mental health component score.

**Figure 8:**
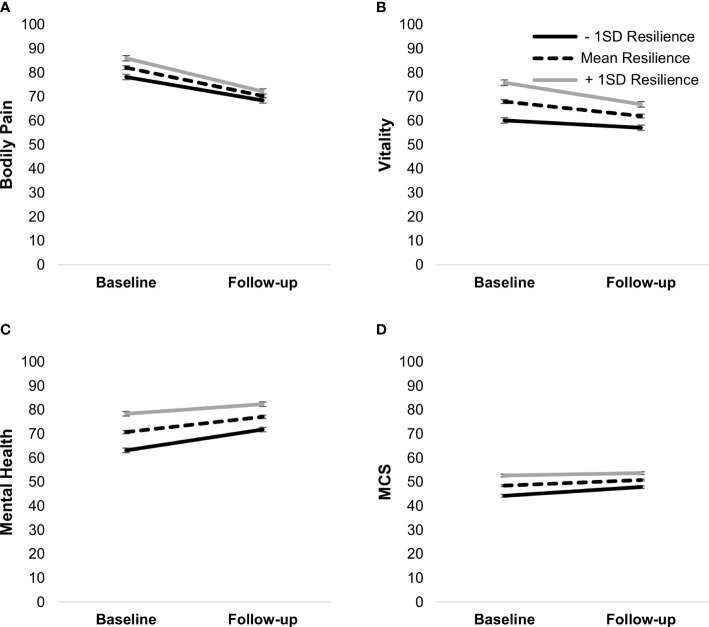
Simple slopes of the moderating effect of baseline resilience on changes in bodily pain **(A)**, vitality **(B)**, mental health **(C)**, and MCS **(D)**. Higher scores on outcome variables indicate better functioning. Error bars show standard errors. MCS, mental health component score.

Similarly, there was a positive association between resilience at baseline and all HRQoL variables (**Table 4**), and resilience at baseline moderated the changes in vitality, bodily pain, mental health, and MCS. Bodily pain and vitality declined in all participants (*p*’s <.01), but the decline was steeper in those with higher baseline resilience. Similarly, mental health increased in all participants (*p*’s <.001), but the increase was steeper for those with lower resilience. MCS increased over time in those with low baseline resilience and those with mean baseline resilience (*p*’s <.001) but stayed the same in those with higher baseline resilience, *t*(758) = 1.66, *p* = .10 (**Figure 8**).

**Table 4:** Resilience at baseline as a covariate to HRQoL (*N =* 760).

	Time			Baseline resilience			Time * Baseline resilience						
	F^a^	*p*		F^a^	*p*		F^a^	*p*		M1^b^	M2^b^	
Physical functioning	137.81	<.001		28.32	<.001		1.23	.27		86.35	80.95	
Role—physical	98.53	<.001		29.03	<.001		1.59	.21		84.24	69.05	
Bodily pain	215.41	<.001		18.53	<.001		6.50	.01		82.09	70.31	
General health	22.89	<.001		106.45	<.001		4.06	.04		71.51	68.46	
Vitality	62.51	<.001		80.52	<.001		15.24	<.001		67.97	61.93	
Social functioning	9.18	.003		59.90	<.001		3.56	.06		85.33	82.66	
Role—emotional	0.00	.95		67.48	<.001		3.14	.08		77.94	77.85	
Mental health	77.33	<.001		132.94	<.001		10.66	.001		70.74	77.07	
PCS^c^	356.76	<.001		14.45	<.001		1.07	.30		54.02	48.73	
MCS^d^	30.47	<.001		112.87	<.001		10.04	.002		48.43	50.75	

HRQoL, health-related quality of life.

a df = 1, 758.

b Estimated marginal means at baseline (M1) and follow-up (M2).

c Physical health component score.

d Mental health component score.

### Sociodemographic variables and health-related quality of life

Participants with a lower socioeconomic status had lower scores on all HRQoL outcomes (all *p*’s <.05). Education level was associated with physical functioning, F(5,748) = 2.56, *p* = .03; individuals with primary education shorter than 9 years had worse physical functioning than those with postsecondary education of two or more years, *t*(748) = 3.44, *p* = .009. Education level was also associated with social functioning, F(5,748) = 2.59, *p* = .02, with individuals with upper secondary education having worse social functioning than those with a PhD degree, *t*(748) = 3.01, *p* = .04. Living arrangement was not associated with any HRQoL outcomes, and sociodemographic variables did not moderate any outcomes (*p*’s <.05).

## Discussion

This study aimed to investigate the process of recovery among BC patients from diagnosis to 1 year post-diagnosis while applying a biopsychosocial perspective and HRQoL as the outcome. Another aim was to study the role of psychological resilience in the process of recovery. In terms of HRQoL, it was noteworthy that mental health improved over time. On the other hand, physical health deteriorated as compared to baseline, especially in terms of physical role limitations, physical functioning, general health, and vitality. Some previous studies done in smaller cohorts showed similar findings in BC patients (55, 56) as well as other types of cancer (57).

The mental HRQoL subscore, however, did not reach the same levels as Swedish norm values (47) even at follow-up, indicating that mental health is not fully recovered after 1 year. It should be noted that baseline data were collected at the time of informing the respondent about the diagnosis and treatment plan, as well as preceded by diagnostic procedures signaling the high possibility of a BC diagnosis. The diagnostic period, with uncertainty and waiting implied, has been identified as highly stressful (58). The lower score and the change in HRQoL therefore likely highlight the toll of the diagnostic process on respondents’ mental health. Thus, alongside the treatment plan addressing breast cancer *per se*, there is a need for an individual rehabilitation plan focusing on areas that hinder their everyday activities. Additionally, the improvement in the mental HRQoL should not be taken as an indication that they do not need psychosocial support.

Nevertheless, the process of recovery is complex, and BC patients represent a diverse group in terms of clinical factors. Higher BC stage and histologic grade seem to be risk factors for low HRQoL. Moreover, most tumor characteristics and treatment modalities assessed in the study were associated with HRQoL, albeit this result should be taken with caution, as significant *p*-values may have been influenced by a large sample size. The finding that symptomatic patients had worse HRQoL than those who discovered their BC through screening is not in line with some previous research (59). It is possible that symptomatic patients experience high levels of uncertainty and shock between discovering a lump and receiving the diagnosis or had more advanced BC. Interestingly, ER-negative tumors seem to be an important risk factor for certain physical HRQoL indicators, whereas HER2-positive tumors seem to be a risk factor for mental HRQoL outcomes. This was the case even after adjusting for radiotherapy and systemic therapy. Important to note is that ER status was not adjusted for endocrine therapy, and HER2 status was not adjusted for antibody therapy, considering that their effects are very hard to distinguish. A potential explanation for HER2 status being associated with mental HRQoL is that the respondents may be informed of the characteristics of their diagnosis and pathological examination of the tumor type, stage, and grade, as well as implications in terms of severity and treatment. Thus, it cannot be ruled out that the association of tumor characteristics with HRQoL is partially a result of understanding the severity of cancer and its treatment. To the best of our knowledge, the moderating effect of tumor characteristics on changes in HRQoL outcomes has not been reported before.

Chemotherapy was associated with slower recovery in various physical HRQoL outcomes, in accordance with previous investigations (10). Aside from chemotherapy, antibody and bisphosphonate therapy were associated with changes in certain HRQoL outcomes, also shown in previous studies (27, 28). Radiotherapy and endocrine therapy had no associations with HRQoL (except for radiotherapy with general health), which is in line with previous research on radiotherapy (10), but not endocrine therapy, which has been associated with many side effects (12, 60). One possible explanation for this is the discontinuation of endocrine treatment, which was not assessed in this study. This may be a limitation; however, adherence to endocrine therapy was found to be high in the Swedish context (61). Moreover, previous studies have shown that most side effects appear within the first few months of treatment, after which quality of life improves (62). It is thus possible that the fluctuations in QoL were not captured in the 1-year follow-up. Endocrine therapy is recommended to be taken for 5 years or more (16), so further research is needed with regard to the long-term impact of endocrine therapy and HRQoL. Additionally, full mastectomy and axillary dissection were associated with worse outcomes as compared to a partial mastectomy and only SLNB, as reported in previous studies (23, 63, 64). The study results indicate that the information about tumor type and treatment plan is vital for outlining the support during treatment and the rehabilitation plan.

The impact of psychological resilience in terms of long-term outcomes indicated that the process of physical and mental health-related recovery was not explained by changes in resilience. Still, resilience may serve as a protective factor for HRQoL, especially at diagnosis and for mental health-related outcomes. Resilience had a positive association with all HRQoL outcomes, implying that patients who had a decline in resilience also had a decline in HRQoL. Changes in resilience did not mediate nor moderate changes in HRQoL. However, although significant, changes in resilience were rather small, suggesting that resilience may not change substantially even during life-altering and highly stressful situations, at least across shorter periods. Whether resilience, as measured by CD-RISC, changes over longer periods remains unanswered. Mediation should be further tested over a longer time, especially since the treatment may be longer in severe cases.

A seemingly surprising finding was that patients with higher baseline resilience had a slower recovery in some outcomes. Comparisons with Swedish norm scores (47) may provide an explanation, as those with higher resilience had better scores than the norm on bodily pain and vitality at baseline and approached the norm values at follow-up. Comparatively, those with lower resilience were below the norm on vitality, and both outcomes were below the norm at follow-up. Therefore, it is possible that there was a floor effect for low resilient patients. Similarly, patients with higher resilience had mental health scores equivalent to the norm at diagnosis and above the norm at follow-up, whereas patients with lower resilience stayed below norm values. Hence, there seems to have been a ceiling effect for those with higher resilience. This suggests that resilience can be protective of one’s physical and mental HRQoL, especially at diagnosis, but only if maintained. Patients with lower resilience may struggle with handling the diagnostic process and take time to recover mentally, requiring additional psychosocial support. Although the role of resilience in the process of recovering HRQoL is not clear, it indicates that it may be a useful measure to identify those in high need already at diagnosis.

Sociodemographic variables further add to BC recovery complexity. The finding that lower SES presents a risk factor for low HRQoL is in line with previous research (10). Lower education level also implied worse physical and social functioning. A study conducted in Sweden found that, although women with a university degree were more likely to be diagnosed with both *in situ* and invasive BC than those who completed less than 9 years of education, they had higher survival, possibly due to a range of behavioral and lifestyle factors (65). Sociodemographic variables did not moderate changes in HRQoL, indicating that the burdens of low SES and education are not diminished throughout the process of BC treatment, with care not being sufficient to overcome preexisting inequalities. Lastly, premenopausal women had better physical HRQoL, but a steeper decline over time, and overall worse mental HRQoL. Previous research suggested younger women with BC are at a higher risk for psychological comorbidity (66) and distress (10), which is in line with them having lower mental health indicators in this study. More severe treatment among younger women (e.g., chemotherapy) may have resulted in a steeper decline in physical HRQoL. Ethnicity was not included in this study. However, previous research conducted in Sweden has found that the effect of country of birth could be explained by education level and SES (cf. 67).

### Strengths, limitations, and future directions

One strength of this study was a relatively large population-based sample, with the majority of newly diagnosed BC patients in the region included. Another strength was having access to a plethora of clinical data. These two factors allowed for an examination of a variety of factors in relation to recovery. Further, measuring HRQoL and resilience immediately at diagnosis, as well as 1 year after, allowed for the assessment of changes in these variables during the most critical period of the BC continuum. Examination of biopsychosocial factors allows for a more comprehensive understanding of the complexity of BC recovery.

This study also had some limitations. First, analyses of attrition showed that non-respondents at follow-up had worse scores on certain physical HRQoL outcomes and lower SES compared to respondents. Thus, scores on physical HRQoL at follow-up could be even lower and the association of SES with HRQoL even stronger. Second, the change in resilience over 1 year was very small, albeit significant. Investigation of the role of resilience on recovery over longer periods is needed. Third, the main and moderating effects of investigated predictors were in most cases small, and significant *p*-values may be in some cases due to a large sample size. In all models, the largest part of the variability in HRQoL was between-patient variability. Therefore, the clinical relevance of these findings should be further explored using qualitative studies, which could provide an understanding of the magnitude and nature of issues experienced by subgroups of patients. Finally, chronic conditions such as lymphedema or radiation-induced brachial plexopathy (RIBP) have not been assessed in this study. The worse HRQoL outcome in those having axillary dissection and those having radiotherapy may well be due to developing lymphedema or RIBP. This study did not go into detail as to the reasons behind the poorer outcome but should be addressed in future studies.

### Conclusion

Although general trends can be observed in relation to physical and mental health-related recovery from BC, the process of recovery is complex. A combination of various factors can implicate a slower recovery and overall worse HRQoL. This includes patients with symptomatic detection, more advanced tumor stage at diagnosis, and ER-negative and HER2-positive tumors, which indicate a more burdening treatment, patients with lower resilience, and lower SES and education. A biopsychosocial approach can comprise a more accurate assessment tool for selecting the most vulnerable patient groups. One year does not seem to be sufficient for recovery from BC, especially in terms of physical recovery, and patients need to be followed up even after most of the treatment has been terminated. Moreover, mental recovery and physical recovery in terms of HRQoL are intertwined. For example, vitality is a measure of both, and resilience is associated with both mental and physical health. Support should thus also be biopsychosocial.

## Data availability statement

The raw data supporting the conclusions of this article will be made available by the authors, without undue reservation.

## Ethics statement

The studies involving human participants were reviewed and approved by Swedish Ethical Review Authority Etikprövningsmyndigheten Box 2110 750 02 Uppsala. The patients/participants provided their written informed consent to participate in this study. Ethical approval numbers obtained for the study are not included in the current version and are as follows: (Dnr 2009/658, 2010/383, 2012/58, 2013/459, 2015/277, 2015/522, 2016/944, 2017/88, 2017/875, 2019-00700, 2019-01351).

## Author contributions

KV conducted conceptualization, methodology, formal analysis, data curation, manuscript preparation, review and editing, and funding acquisition. CB undertook conceptualization, resources, manuscript review, and funding acquisition. P-OB conducted methodology and formal analysis, data curation, manuscript review and editing, and supervision. CH did the investigation, manuscript review and editing, and project administration. PJ did the conceptualization, investigation, resources, manuscript review and editing, supervision, funding acquisition and conception of the study integrating biology/clinical parameters. CR undertook the investigation, data curation, manuscript review and editing, and project administration. LR participated in methodology, validation, resources, manuscript review and editing, supervision, funding acquisition and conception of the study integrating biology/clinical parameters. IRH did the conceptualization, methodology, validation, resources, manuscript review and editing, supervision, project administration, and funding acquisition. All authors contributed to the article and approved the submitted version.

## Funding

This study was supported by grants from the European Research Council (EU-MSCA-COFUND 754299 CanFaster), the Department of Psychology, Lund University, the Faculty of Medicine, Lund University, the Mats Paulsson Foundation, and the CREATE Health Cancer Center. SCAN-B was funded by the Swedish Cancer Society, the Mrs. Berta Kamprad Foundation, the Lund-Lausanne L2-Bridge/Biltema Foundation, the Mats Paulsson Foundation, and Swedish governmental funding (ALF), grand number 2018-40304.

## Acknowledgments

The authors acknowledge the SCAN-B steering group, the SCAN-B Resilience study group, the Regional Cancer Center South, the staff at the central SCAN-B laboratory at the Division of Oncology, Lund University, the Swedish national breast cancer quality registry (NKBC), and the South Swedish Breast Cancer Group (SSBCG) for all their support. The authors also thank all patients who took part in the study as well as the hospital staff who helped with recruitment and data collection.

## Conflict of interest

The authors declare that the research was conducted in the absence of any commercial or financial relationships that could be construed as a potential conflict of interest.

## Publisher’s note

All claims expressed in this article are solely those of the authors and do not necessarily represent those of their affiliated organizations, or those of the publisher, the editors and the reviewers. Any product that may be evaluated in this article, or claim that may be made by its manufacturer, is not guaranteed or endorsed by the publisher.
