# Supplementary material for: One-year recovery from breast cancer: Importance of tumor and treatment-related factors, resilience, and sociodemographic factors for health-related quality of life
Source: Front Oncol. 2022 Aug 16;12:891850. doi: 10.3389/fonc.2022.891850 (PMC9425776; doi:10.3389/fonc.2022.891850)
Supplement: Supplementary file 1 [file Table_1.docx]

|  | Var | 1 | 2 | 3 | 4 | 5 | 6 | 7 | 8 | 9 | 10 | 11 | 12 | 13 | 14 | 15 | 16 | 17 | 18 | 19 | 20 | 21 | 22 |
| --- | --- | --- | --- | --- | --- | --- | --- | --- | --- | --- | --- | --- | --- | --- | --- | --- | --- | --- | --- | --- | --- | --- | --- |
| BL |  |  |  |  |  |  |  |  |  |  |  |  |  |  |  |  |  |  |  |  |  |  |  |
|  | 1.CD |  |  |  |  |  |  |  |  |  |  |  |  |  |  |  |  |  |  |  |  |  |  |
|  | 2.PF | .20*** |  |  |  |  |  |  |  |  |  |  |  |  |  |  |  |  |  |  |  |  |  |
|  | 3.RP | .21*** | .58*** |  |  |  |  |  |  |  |  |  |  |  |  |  |  |  |  |  |  |  |  |
|  | 4.BP | .19*** | .57*** | .51*** |  |  |  |  |  |  |  |  |  |  |  |  |  |  |  |  |  |  |  |
|  | 5.GH | .37*** | .45*** | .41*** | .43*** |  |  |  |  |  |  |  |  |  |  |  |  |  |  |  |  |  |  |
|  | 6.VT | .35*** | .42*** | .52*** | .50*** | .56*** |  |  |  |  |  |  |  |  |  |  |  |  |  |  |  |  |  |
|  | 7.SF | .28*** | .30*** | .46*** | .35*** | .43*** | .60*** |  |  |  |  |  |  |  |  |  |  |  |  |  |  |  |  |
|  | 8.RE | .27*** | .33*** | .55*** | .34*** | .38*** | .57*** | .59*** |  |  |  |  |  |  |  |  |  |  |  |  |  |  |  |
|  | 9.MH | .37*** | .16*** | .28*** | .21*** | .44*** | .62*** | .62*** | .50*** |  |  |  |  |  |  |  |  |  |  |  |  |  |  |
|  | 10.PC | .15*** | .82*** | .70*** | .75*** | .53*** | .38*** | .20*** | .16*** | -.07 |  |  |  |  |  |  |  |  |  |  |  |  |  |
|  | 11.MC | .36*** | .12** | .35*** | .21*** | .44*** | .72*** | .77*** | .78*** | .89*** | -.09* |  |  |  |  |  |  |  |  |  |  |  |  |
| FU |  |  |  |  |  |  |  |  |  |  |  |  |  |  |  |  |  |  |  |  |  |  |  |
|  | 12.CD | .66*** | .20*** | .23*** | .16*** | .32*** | .32*** | .23*** | .24*** | .30*** | .16*** | .30*** |  |  |  |  |  |  |  |  |  |  |  |
|  | 13.PF | .15*** | .77*** | .48*** | .49*** | .41*** | .38*** | .27*** | .30*** | .15*** | .66*** | .13*** | .23*** |  |  |  |  |  |  |  |  |  |  |
|  | 14.RP | .12** | .36*** | .35*** | .33*** | .34*** | .37*** | .30*** | .31*** | .21*** | .35*** | .26*** | .22*** | .56*** |  |  |  |  |  |  |  |  |  |
|  | 15.BP | .08* | .39*** | .32*** | .48*** | .37*** | .36*** | .30*** | .25*** | .19*** | .43*** | .21*** | .17*** | .56*** | .58*** |  |  |  |  |  |  |  |  |
|  | 16.GH | .27*** | .42*** | .37*** | .40*** | .63*** | .49*** | .33*** | .33*** | .30*** | .46*** | .32*** | .41*** | .54*** | .53*** | .55*** |  |  |  |  |  |  |  |
|  | 17.VT | .20*** | .35*** | .35*** | .40*** | .44*** | .56*** | .37*** | .35*** | .34*** | .35*** | .40*** | .37*** | .52*** | .62*** | .60*** | .67*** |  |  |  |  |  |  |
|  | 18.SF | .18*** | .33*** | .36*** | .32*** | .38*** | .47*** | .43*** | .35*** | .35*** | .29*** | .40*** | .29*** | .49*** | .62*** | .53*** | .58*** | .70*** |  |  |  |  |  |
|  | 19.RE | .20*** | .25*** | .31*** | .25*** | .29*** | .39*** | .34*** | .35*** | .29*** | .21*** | .36*** | .32*** | .37*** | .60*** | .40*** | .46*** | .57*** | .65*** |  |  |  |  |
|  | 20.MH | .29*** | .21*** | .29*** | .28*** | .37*** | .49*** | .39*** | .35*** | .48*** | .16*** | .49*** | .47*** | .33*** | .43*** | .40*** | .57*** | .70*** | .66*** | .60*** |  |  |  |
|  | 21.PC | .10** | .61*** | .41*** | .49*** | .46*** | .36*** | .36*** | .27*** | .11** | .60*** | .13*** | .17*** | .82*** | .76*** | .78*** | .67*** | .58*** | .49*** | .29*** | .23*** |  |  |
|  | 22.MC | .26*** | .15*** | .28*** | .23*** | .34*** | .50*** | .41*** | .37*** | .44*** | .12*** | .49*** | .43*** | .26*** | .48*** | .37*** | .54*** | .75*** | .79*** | .83*** | .89*** | .18*** |  |
| *M* |  | 70.73 | 85.92 | 83.52 | 82.04 | 71.30 | 67.71 | 85.03 | 77.38 | 70.75 | 53.89 | 48.32 | 69.18 | 80.76 | 68.46 | 70.21 | 68.30 | 61.83 | 82.27 | 77.24 | 76.96 | 48.66 | 50.62 |
| *SD* |  | 12.59 | 18.22 | 32.99 | 20.42 | 18.95 | 22.52 | 21.39 | 36.44 | 20.68 | 8.13 | 11.81 | 13.62 | 20.17 | 40.86 | 22.90 | 21.40 | 23.77 | 24.22 | 37.10 | 18.25 | 9.15 | 11.32 |

***Supplementary Material***

**Supplementary Table.** Pearson’s *r*, between CD-RISC and SF-36 subscores at baseline and follow-up, means, and standard deviations (*N* = 760)

Note. * *p*<.05, ** *p*<.01, *** *p*<.001. BL = Baseline; FU = Follow-up; CD = Resilience; PF = Physical functioning; RP = Role limitations due to physical problems; BP = Bodily pain; GH = General health; VT = Vitality; SF = Social functioning; RE = Role limitations due to emotional problems; MH = Mental health; PC = Physical health summary score; MCS = Mental health summary score
